# Structure of the AvrBs3–DNA complex provides new insights into the initial thymine-recognition mechanism

**DOI:** 10.1107/S0907444913016429

**Published:** 2013-08-15

**Authors:** Stefano Stella, Rafael Molina, Igor Yefimenko, Jesús Prieto, George Silva, Claudia Bertonati, Alexandre Juillerat, Phillippe Duchateau, Guillermo Montoya

**Affiliations:** a Structural Biology and Biocomputing Programme, Spanish Cancer Research Centre (CNIO), Melchor Fernandez Almagro, 28029 Madrid, Spain; b Cellectis, 8 Rue de la Croix Jarry, 75013 Paris, France

**Keywords:** gene targeting, protein–DNA interaction, genetics

## Abstract

Transcription activator-like effectors contain a DNA-binding domain organized in tandem repeats. The repeats include two adjacent residues known as the repeat variable di-residue, which recognize a single base pair, establishing a direct code between the dipeptides and the target DNA. This feature suggests this scaffold as an excellent candidate to generate new protein–DNA specificities for biotechnological applications. Here, the crystal structure of AvrBs3 (residues 152–895, molecular mass 82 kDa) in complex with its target DNA sequence is presented, revealing a new mode of interaction with the initial thymine of the target sequence, together with an analysis of both the binding specificity and the thermodynamic properties of AvrBs3. This study quantifies the affinity and the specificity between AvrBs3 and its target DNA. Moreover, *in vitro* and *in vivo* analyses reveal that AvrBs3 does not show a strict nucleotide-binding preference for the nucleotide at the zero position of the DNA, widening the number of possible sequences that could be targeted by this scaffold.

## Abstract 2

The crystal structure of the AvrBs3–DNA complex is reported.

## 1. Introduction

Transcription activator-like effectors (TALEs) compose a family of virulence proteins that act as transcriptional activator factors in plant cells (Boch & Bonas, 2010 ▶). TALEs are organized into three different domains: an N-terminal region involved in protein translocation by the bacterial secretion system (Bogdanove *et al.*, 2010 ▶), a central DNA-binding domain and a C-terminal region that contains both the nuclear localization signal sequence and an acidic transcriptional activation region. The central DNA-binding domain is composed of an array of tandem repeats which recognizes the DNA target. The repeats contain a conserved sequence of 30–­42 residues constituting a new DNA-binding motif (Boch & Bonas, 2010 ▶). The number of repeats in the DNA-binding domain of the TALE proteins ranges between 1.5 and 33.5; the last repeat of the DNA-binding domain only contains half of the residues (Boch & Bonas, 2010 ▶; Boch *et al.*, 2009 ▶). It is most likely that the smaller TALEs are nonfunctional, as a minimum number of 6.5 repeats is needed to induce target-gene expression (Boch *et al.*, 2009 ▶). The amino-acid sequence of each repeat is well conserved, with the exception of two contiguous amino acids at positions 12 and 13 known as the repeat variable di-residue (RVD). The DNA bases recognized by each repeat are specified by the amino-acid sequence of the RVDs, establishing a direct code between these pairs of amino acids in each repeat and the nucleotides in the target sequence (Boch *et al.*, 2009 ▶; Moscou & Bogdanove, 2009 ▶).

More than 20 different RVDs have been identified in the different TALEs examined to date (Cong *et al.*, 2012 ▶). However, some of the dipeptides can bind several bases, promoting a degeneration of the protein–DNA recognition code (Boch *et al.*, 2009 ▶; Streubel *et al.*, 2012 ▶). Other residues outside the RVD dipeptides do not show a significant effect on base-pair specificity (Moscou & Bogdan­ove, 2009 ▶; Morbitzer *et al.*, 2010 ▶). Structural data showed that only the residue in position 13 of the RVD makes specific contacts for target DNA recognition, while the amino acid at position 12 seems to stabilize the repeat (Deng *et al.*, 2012 ▶; Mak *et al.*, 2012 ▶). The simple RVD nucleotide code allows the design of new TALEs generated with new repeat combinations. The assembly of several repeats in redesigned TALEs recognizing new DNA targets has confirmed the modularity of these DNA-binding domains and their use in biotechnological applications (Bogdanove & Voytas, 2011 ▶; Miller *et al.*, 2011 ▶). The TALE–DNA interaction orients the protein repeats in the N-terminal to C-terminal direction contacting the 5′–3′-sense DNA strand. All of the natural targets contain a 5′ T (also known as T_0_) preceding the recognized DNA, which has been reported to be important for TALE activity (Bogdanove & Voytas, 2011 ▶).

Here, we present the crystal structure of the DNA-binding region of AvrBs3 from *Xanthomonas campestris* bound to its target sequence present in the pepper Bs3 promoter, including the N-terminal region interacting with the initial thymine. The structure reveals a new mode of interaction of this domain with T_0_. These data, together with analysis of the protein–DNA binding *in vitro* and the activity *in vivo*, suggest that AvrBs3 is able recognize its target despite the base at position zero.

## 2. Materials and methods

### 2.1. Protein expression and purification

The AvrBs3 used in this study contains some modifications that seem to improve protein expression without altering specificity. The NI RVDs were used to target adenine (except for the first repeat) and one of the NG RVDs differs from the wild-type sequence. The coding cDNA for AvrBs3 was cloned into a pET-derived vector and transformed into *Escherichia coli* BL21. The cells were grown in LB medium at 310 K and protein expression was induced with 1 m*M* IPTG for 2 h when the culture reached an OD at 600 nm of 1. The induced cells were disrupted by sonication in buffer *A* (50 m*M* HEPES pH 8.0, 150 m*M* NaCl, 0.5 m*M* imidazole, 0.5 m*M* TCEP). Cell debris was removed by centrifugation and the supernatant was loaded onto an Ni–NTA (GE Healthcare) column. After extensive column washing, the protein was eluted with a linear gradient to 500 m*M* imidazole. Fractions containing the protein were pooled and loaded onto a heparin column (GE Healthcare) equilibrated in buffer *B* (50 m*M* HEPES pH 8.0, 150 m*M* NaCl, 0.5 m*M* TCEP). The protein was eluted using a linear gradient to 1 *M* NaCl. The fractions containing the protein were pooled and loaded onto a Superdex 200 (GE Healthcare) gel-filtration column equilibrated in buffer *A*. Protein fractions were pooled and stored at 193 K.

### 2.2. Crystallization, data collection, structure solution, model building and refinement

**Figure 1:**
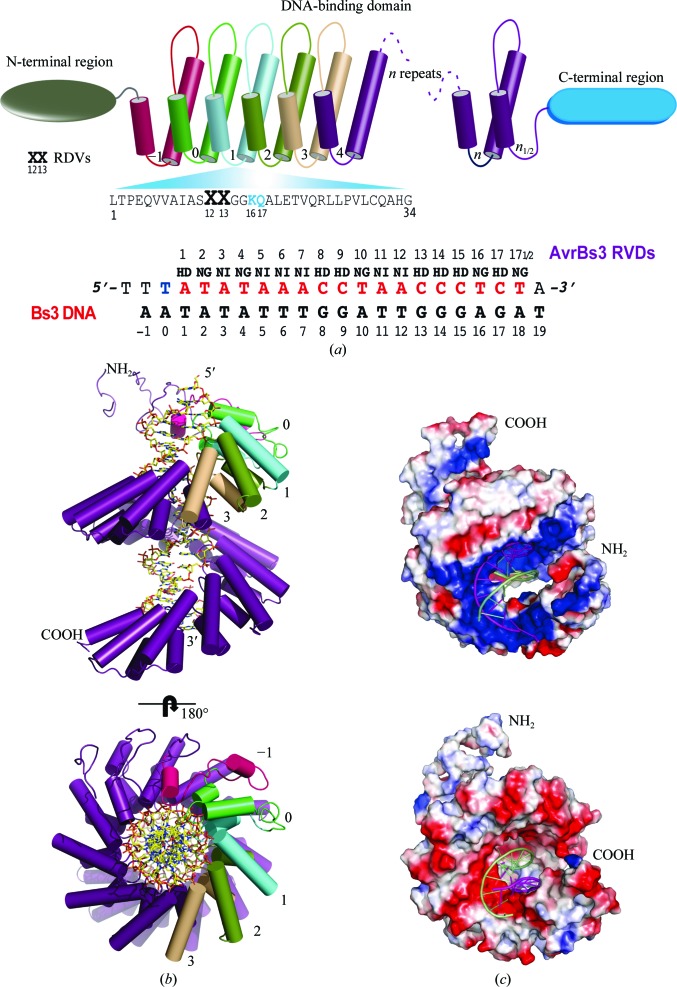
Crystal structure of the AvrBs3–DNA complex. (*a*) Sketch of the TALE domain structure. The N-terminus is coloured green and the C-terminus is coloured blue. The central DNA-binding domain contains the RVDs involved in DNA recognition. The sequence of the first repeat (cyan) of AvrBs3 is shown, indicating the position of each amino acid in the repeat. The sequence of the oligonucleotide used in crystallization and the different dipeptides is depicted below. (*b*) Cartoon representation of the protein–DNA structure perpendicular to the DNA target (upper panel) and along the DNA helix (lower panel). The helices of AvrBs3 are shown as cylinders and repeats −1, 0, 1, 2 and 3 are coloured red, lime, cyan, green and beige, respectively, to indicate the initial DNA domain building blocks. The duplex oligonucleotide is represented in stick mode and the sense-strand sequence is indicated with the corresponding dipeptide for each nucleotide. The dipeptides are represented using the single-letter amino-acid code. (*c*) Electrostatic surface representation of the AvrBs3 protein in complex with its DNA target. The upper panel depicts the electropositive strip (blue) on the superhelical arrangement running from the amino-terminus to the carboxyl-terminus. The electronegative strip (red) running along the protein in the opposite side is depicted in the lower panel. The sense strand is coloured magenta and the antisense strand is coloured green.

The purified AvrBs3 DNA-binding domain was crystallized in complex with a 21-base-pair DNA duplex containing the target sequence of the pepper Bs3 promoter (see Fig. 1 ▶ *a* for the oligonucleotide sequence and Supplementary Fig. S1^1^ for the protein sequence). The 21 bp DNAs (IDT) for crystallo­graphy were annealed by slow-cooling in 25 m*M* HEPES pH 8.0, 150 m*M* NaCl at a final duplex concentration of 0.5 m*M*. A 1.2 molar excess of TALE AvrBS3 relative to the DNA was incubated on ice for 10 min at a protein–DNA concentration of 7 mg ml^−1^ in a solution consisting of 25 m*M* HEPES pH 8, 150 m*M* NaCl, 0.2 m*M* TCEP. The complex was dialyzed against 20 m*M* MES pH 6.0, 100 m*M* NaCl, 5 m*M* MgCl_2_ at 277 K for 1 h. The crystallization was performed with 0.8 µl sitting drops using a Cartesian 4000 XL robot. Optimal quality crystals were grown at 298 K using a 1:1 ratio of complex solution and reservoir solution [100 m*M* MES pH 6.5, 5–­15%(*v*/*v*) PEG 3350, 5–15%(*v*/*v*) 2-propanol]. Crystals grown after 2–3 d were cryoprotected by adding 30%(*v*/*v*) 2-­propanol to the mother liquor and were flash-cooled in liquid nitrogen. Diffraction data were collected at 100 K using synchrotron radiation on the PXI-XS06 beamline at SLS, Villigen, Switzerland. Data-processing and scaling were accomplished with *XDS* (Kabsch, 2010 ▶; Table 1 ▶). Initial phases were obtained by combining information from a Ta_6_Br_12_-cluster single-wavelength anomalous diffraction (SAD) data set and molecular-replacement information obtained from a previous partial model obtained using *Phaser* (McCoy *et al.*, 2007 ▶) (see Table 1 ▶ and Supplementary Fig. S2). The anomalous Patterson showed the presence of two possible sites, and two Ta_6_Br_12_ clusters were found using the *SHELX* package (Sheldrick, 2008 ▶). The search model for molecular replacement was based on a polyalanine backbone of three RVD repeats derived from PDB entry 3v6t (Deng *et al.*, 2012 ▶). The initial molecular-replacement phases displayed density that was not well defined in several protein regions. The combination of the heavy-atom cluster phases with the molecular-replacement solution yielded an improved electron-density map. These initial phases were extended to 2.55 Å resolution using a higher resolution native data set with the *AutoBuild* routine in *PHENIX* (Adams *et al.*, 2010 ▶). The structure was built and subjected to iterative cycles of model building with *Coot* (Emsley *et al.*, 2010 ▶) and refinement by combining *REFMAC* (Murshudov *et al.*, 2011 ▶) and *PHENIX* (Adams *et al.*, 2010 ▶). Identification and analysis of the protein–DNA hydrogen bonds and van der Waals contacts was performed with the *Protein Interfaces, Surfaces and Assemblies* service (*PISA*) at the European Bioinformatics Institute (http://www.ebi.ac.uk/msdsrv/prot_int/pistart.html).

### 2.3. Fluorescence anisotropy

The dissociation constants between the TALE protein and DNA were estimated from the change in fluorescent polarization upon protein addition using oligonucleotides that were 6-FAM-labelled at the their 5′-end. The optimal concentration of the 6-FAM-DNAs was determined empirically by measuring the fluorescence polarization of serially diluted 6-­FAM-DNA samples (Molina *et al.*, 2012 ▶). The concentration of the 6-FAM-labelled DNAs ranged between 20 and 40 n*M* and that of the TALE protein was increased to 1000 n*M*. Both proteins and DNAs were dialyzed in buffer consisting of 25 m*M* HEPES pH 8, 150 m*M* NaCl, 0.2 m*M* TCEP. After incubation at 298 K for 10 min, the fluorescence polarization was measured in a black 96-well assay plate with a Wallac 1420 VICTOR2 multilabel counter (PerkinElmer). The fitting of the data and the *K*_d_ calculations were performed as described in Molina *et al.* (2012 ▶). For the competitive binding assay, the concentration of the 24 bp nonspecific DNA duplex (5′-­TCAGACTTCTCCACAGGAGTCAGA-3′) was 100 µ*M*.

### 2.4. Isothermal titration calorimetry assays

Isothermal titration calorimetry (ITC) experiments were conducted at 298 K using a MicroCal ITC200 instrument (Microcal GE Healthcare, UK). The buffer consisted of 25 m*M* HEPES pH 8, 150 m*M* NaCl, 0.2 m*M* TCEP. To ensure minimal buffer mismatch, protein and DNA samples were dialyzed against the same buffer. The syringe for the ligand contained DNA duplexes in the concentration range 0.06–0.2 m*M*. The thermostatic cell contained the TALE protein in the concentration range 0.006–0.02  m*M*. Competitive binding studies were carried out using the strong-binding ligand A (target DNA) as the injectant, with the solution in the cell containing the second competitive ligand B (competitor DNA) as well as the TALE (T). This system then has two equilibria that are displaced with each injection:
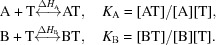



The values of *K*_B_ and Δ*H*_B_ for the competing ligand were first measured in a conventional ITC experiment, and these parameter values are entered as known parameters when determining *K*_A_ from the results of the competition experiment. For the competition experiment, the total concentration of competitor [B]_tot_ was calculated using the formula
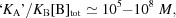
where ‘*K*_A_’ is the estimated association constant of the TALE for the target DNA obtained in the best concentration range (10^5^–10^8^ *M* ^−1^) for measurements for ITC. The thermostatic cell contains the TALE protein in the concentration range 0.006–0.01 m*M* and competitor DNA at a concentration of 0.005 m*M*. The syringe for the ligand contained the DNA duplex in the concentration range 0.06–0.1 m*M*. The experiments consisted of a series of 4 µl injections of DNA into 200 µl protein solution in the thermostatic cell with an initial delay of 60 s, a 4 s duration of injection and a spacing between injections of 180 s. The corrected binding isotherms were fitted using a single-site and competitive-binding model nonlinear least-squares analysis with the *Origin* 7.0 software (MicroCal) to obtain values of the equilibrium binding constant (*K*_A_), stoichiometry (*n*) and enthalpy changes (Δ*H*) and the *T*Δ*S* associated with DNA binding. The *K*_d_ was the inverse of the calculated *K*_A_ and the associated error was estimated using an error-propagation calculator (http://laffers.net/tools/error-propagation-calculator/).

### 2.5. Mating of TALE nuclease (TALEN) expressing clones and screening in yeast

The yeast strain expressing the TALEN to be assayed is mated with a strain harbouring a reporter plasmid containing the chosen target, which is flanked by overlapping truncated *lacZ* genes (LAC and ACZ). Upon target cleavage, tandem-repeat recombination restores a functional *lacZ* gene that can be monitored using standard methods. TALENs were gridded on nylon filters covering YPD plates using a high gridding density (about 20 spots cm^−2^). A second gridding process was performed on the same filters to spot a second layer consisting of reporter-harbouring yeast strains for each target. Membranes were placed on solid agar YPD-rich medium and incubated at 303 K overnight to allow mating. Next, the filters were transferred to a synthetic medium lacking leucine and tryptophan with galactose (2%) as a carbon source and were incubated for 5 d at 310 K to select for diploids carrying the expression and target vectors. After 5 d, filters were placed on solid agarose medium with 0.02%(*w*/*v*) X-Gal in 0.5 *M* sodium phosphate buffer pH 7.0, 0.1%(*w*/*v*) SDS, 6% dimethyl­formamide (DMF), 7 m*M* β-mercaptoethanol, 1%(*w*/*v*) agarose and incubated at 310 K to monitor β-galactosidase activity. Results were analysed by scanning and quantification was performed. β-Galactosidase activity is directly associated with the efficiency of homologous recombination. Experiments using several purified I-CreI mutants with various recombination activities in yeast have shown that the recombination efficiency quantified in yeast (Afilter value) is correlated with the cleavage activity *in vitro* (Arnould *et al.*, 2007 ▶; Grizot *et al.*, 2009 ▶).

## 3. Results and discussion

### 3.1. Overall structure of the AvrBs3–DNA complex

The structure of the protein–DNA complex was solved by combining a Ta_6_Br_12_ SAD data set and a molecular-replacement solution (Table 1 ▶). The model was refined to 2.55 Å resolution. The crystallized protein includes residues 152–895 (Supplementary Fig. S1) of AvrBs3 and a 21-base double-strand oligonucleotide with a T overhang at the 5′-end of the sense strand (Figs. 1 ▶ *a* and 1 ▶ *b*), displaying a relatively unperturbed B-form DNA with an overall wider major groove (Supplementary Fig. S2). The electron density for the 30 amino-terminal and carboxyl-terminal residues is fuzzy owing to protein flexibility. However, the quality of the electron density is excellent from the first repeat until the middle of repeat 17 in residue 830; in the N-terminal region the electron density is defined such that side chains can be observed from residue 230 onwards. The superhelical arrangement of the 17.5 AvrBs3 repeats is intimately engaged in binding the major-groove nucleotides of the DNA molecule (Figs. 1 ▶ *b* and 2 ▶). All of the repeats in the DNA-bound AvrBs3 structure form highly similar two-helix bundles (Fig. 1 ▶ *b*). The helices span positions 3–11 and 14–33 in the repeat, locating the RVD (positions 12 and 13; see Fig. 1 ▶ *a*) in the loop that joins them. The proline at position 27 creates a kink in the second helix that appears to be critical for sequential packing and association of tandem repeats with the DNA double helix. The protein shows a left-handed packing of the consecutive helices within and between the individual repeats.

### 3.2. Protein–DNA interaction

Interestingly, an electropositive strip runs along one side of the superhelical AvrBs3 arrangement (Deng *et al.*, 2012 ▶) and an electronegative strip is observed on the opposite side (Fig. 1 ▶ *c*). This positive polar band is built by a lysine at position 16 in each repeat and involves nonspecific interactions with the phosphate backbone of the DNA sense strand, whereas the negative band, built mainly by the glutamates at position 4 of the repeats with the collaboration of some of the aspartates at position 13, lies in the neighbourhood of the antisense DNA strand (Fig. 1 ▶ *c*). The arrangement of these polar bands along the protein suggests a possible mechanism to facilitate the recognition of the nucleotides in the sense strand by the RVDs while avoiding interference from the nucleotides in the other strand. In fact, the antisense strand does not display contacts with the protein (Supplementary Fig. S3).

The sequence-specific contacts of TALE AvrBs3 with the DNA are exclusively made by the residue at position 13 in each RVD interacting with the corresponding base on the sense strand (Supplementary Fig. S4). In contrast, the side chain of the residue at position 12 of each RVD contacts the backbone carbonyl O atom at position 8 in each repeat, constraining the RVD-containing loop. Additionally, the positions within the core of individual repeats are occupied entirely by small aliphatic residues, whereas several positions in the interface between repeats correspond to polar residue pairs.

### 3.3. Dipeptide–DNA interaction

**Figure 2:**
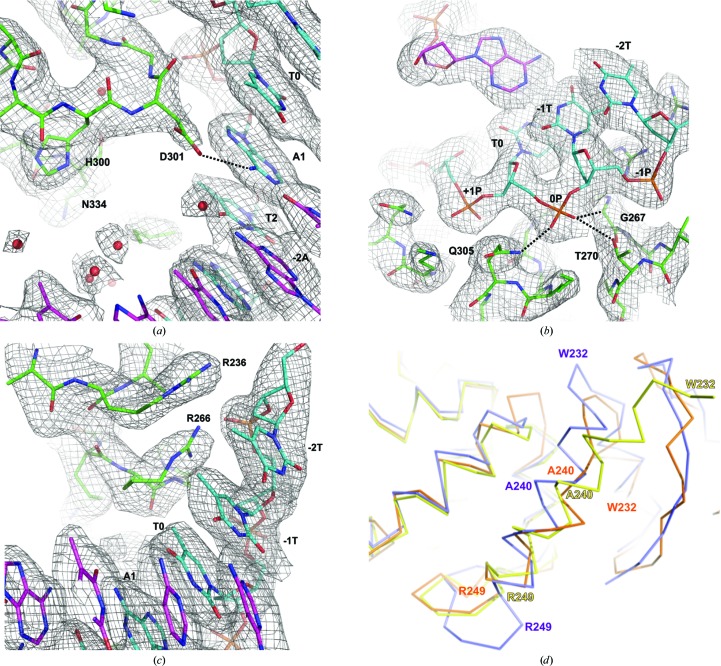
Recognition of the A_1_ and T_0_ positions by AvrBs3. (*a*) The initial HD dipeptide associated with the first adenine in the target sequence. Asp301 forms a hydrogen bond to the amino group of the base. (*b*) The interactions of T_0_ with the N-terminal region of AvrBs3. The phosphate of the nucleotide interacts with Thr270, Gln305 and Gly268 through hydrogen bonds. (*c*) Detailed view of T_0_ interacting with Arg236 and Arg266 through nonpolar van der Waals interactions. The map in all of the figures is a 2*F*_o_ − *F*_c_ σ_A_-weighted map contoured at 1.0σ. (*d*) Superposition of the PthXo1 (purple; PDB entry 3ugm; Mak *et al.*, 2012 ▶), dHax3 (yellow; PDB entry 3v6t; Deng *et al.*, 2012 ▶) and AvrBs3 (orange) protein structures. The DNA moiety is omitted for clarity. Differences among the three TALE structures in the N-terminal region (including the 0 and −1 repeats) can be observed (see *Results and discussion* [Sec sec3] and Supplementary Fig. S5).

The AvrBs3–DNA structure displays seven HD RVDs. The pair in the first repeat contains a unique HD associated with adenine (Fig. 2 ▶ *a*). The other HD dipeptides interact with cytosines along the target sequence. The rest of the adenines are associated with NI dipeptides and the four thymines interact with NG dipeptides (Fig. 1 ▶ *a*). The observed contacts for each repeat (Supplementary Figs. S3 and S4) shed light on the molecular basis of their different specificity and fidelity, which has been described *via* computational and genetic analyses (Moscou & Bogdanove, 2009 ▶; Streubel *et al.*, 2012 ▶). The HD RVD contacting A_1_ displays a hydrogen bond (3.01 Å) between the side chain of Asp301 in the first RVD and the NH_2_ group of the base. The interaction is the same as when the HD recognizes a cytosine. In contrast, His300 in the initial RVD does not interact with the DNA and its side chain contacts the main-chain backbone of the following repeat, stabilizing the interface between the first and the second repeats (Fig. 2 ▶ *a*).

The rest of the HD RVDs show the aspartate residues associated with the NH_2_ group of the cytosines through hydrogen bonds ranging from 2.95 to 3.5 Å in length along the sense DNA strand. Contacts between cytosine and acidic side chains exclude alternative base recognition *via* steric and electrostatic clashes (Rohs *et al.*, 2010 ▶). The NI dipeptide exhibits an unusual interaction pattern with the other adenines. The aliphatic side chain of the isoleucine residue makes nonpolar van der Waals interactions with the purine ring, and the asparagine residues play a role similar to that of the histidine in the HD dipeptides, stabilizing the inter-repeat interaction. The fact that five of them are grouped in two regions containing three and two consecutive adenines extends these two similar interaction areas along the DNA target. Finally, the NG repeats associate with the thymines through nonpolar van der Waals interactions of the glycine main chain with the methyl group of the base. This interaction is barely observed in T_18_ owing to disorder of the last repeat.

The TALE repeats seem to be organized into two regions interacting with the sense stand, whereas the antisense strand does not display any protein contacts. The first region, which is involved in indirect readout, is composed of a lysine and a glutamine at positions 16 and 17 of the repeat and interacts with the sense-strand DNA backbone (Fig. 1 ▶ *a*). This arrangement is conserved both in PthXo1 and dHax3. The second region involves the RVDs, which interact directly with the bases. Among the structurally characterized RVDs in the different structures available, NN and HD form hydrogen bonds to their target nucleotides, while NI and NG associate with their target bases through van der Waals interactions. Thus, the energy involved in the different interactions establishes a hierarchy between the different RVDs (Streubel *et al.*, 2012 ▶). Nevertheless, even the HD dipeptide, which shows a preferential interaction with cytosine and is one of the energetically selective RVDs, can accommodate adenine through a hydrogen bond to its NH_2_ group (Fig. 2 ▶ *a*), suggesting a certain promiscuity of the dipeptides even in the energetically more selective RVDs. Although an RVD–nucleotide preference exists, the dipeptide–base interactions do not build a strict binary code since the same dipeptide can interact with different bases, promoting a certain degree of degeneration of the protein–DNA recognition. Therefore, the energetic contribution of the different dipeptides during binding seems to be crucial to generate a selective TALE, suggesting that TALE specificity would depend on the energy balance between the region involved in indirect readout (Fig. 1 ▶ *c*) and the contribution of the RVD.

### 3.4. The T_0_ recognition

A possible limitation to engineering new recognition sequences in this scaffold arises from the presence of a T at the zero position of the target DNA at the 5′-end. This base interacts with the N-terminus of the protein and appears to be critical for the TALE–DNA interaction (Boch *et al.*, 2009 ▶; Bogdanove & Voytas, 2011 ▶). Although the crystal structure of the TALE dHax3 DNA-binding domain lacks the N-terminal domain (Deng *et al.*, 2012 ▶), the structure of the PthXo1–DNA complex suggests that the conserved Trp232 is involved in the recognition mechanism of the T_0_ at the 5′-end (Mak *et al.*, 2012 ▶). However, this residue does not display direct inter­actions with the base. The N-terminal region of AvrBs3 reveals that two degenerate repeats seem to cooperate to interact with the conserved thymine that precedes the RVD-specified sequence (Fig. 2 ▶ *b*). We termed these the 0 and −1 repeats (Fig. 1 ▶ *b*, Supplementary Fig. S1) composed of residues 225–254 and 255–288, respectively. They contain an arginine residue (Arg266 and Arg236, respectively) at position 13 that interacts with the DNA (Fig. 2 ▶ *c*). The side chains of these residues converge near the adjacent T_−1_ and T_0_ bases, contacting the methyl groups of these bases through van der Waals interactions. Moreover, the side chains of Thr270, Gln305 and Gly267 are involved in a network of hydrogen bonds surrounding the phosphate of T_0_. In contrast to PthXo1, the Trp232 in AvrBs3 is located four positions away from the DNA. This difference between PthXo1 and AvrBs3 arises from a different conformation in the protein section preceding these residues, which is more elongated in the AvrBs3 structure, displacing Trp232 away from the DNA (Fig. 2 ▶ *d* and Supplementary Fig. S5).

These differences could arise from the intrinsic flexibility of the TALE repeats, which seem to display a large conformational change (Murakami *et al.*, 2010 ▶). This flexibility has also been observed in assemblies of these repeats composing a DNA-binding domain in the absence of nucleic acid by SAXS (Murakami *et al.*, 2010 ▶). In addition, the crystal structure of dHax3 without its target DNA (Deng *et al.*, 2012 ▶) shows an elongated shape, suggesting that the protein conformation is adjusted to the target DNA and is stabilized upon nucleic acid binding. This flexible behaviour of the TALEs could facilitate DNA binding. On the other hand, the fact that the crystallized proteins lack a section of the N-terminal sequence could favour these conformational changes.

### 3.5. TALE–DNA binding and thermodynamics

**Figure 3:**
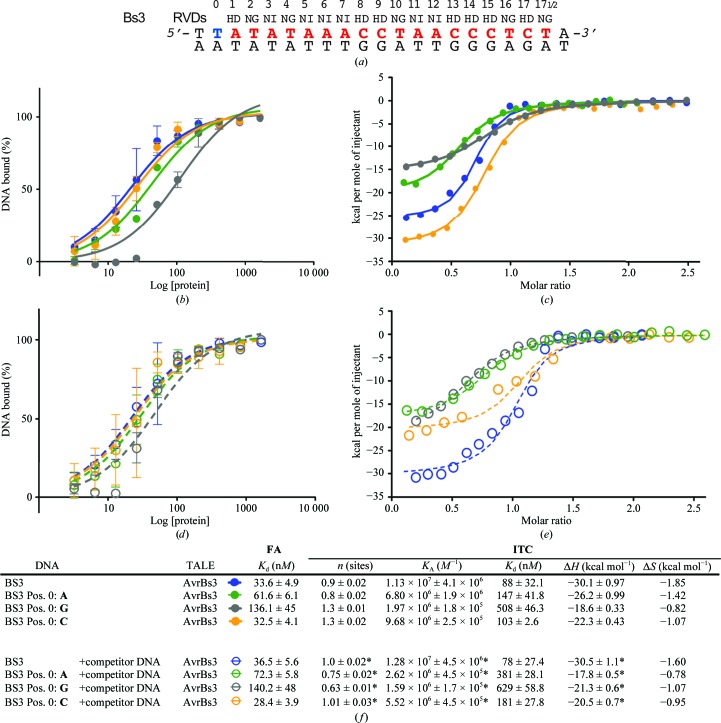
Thermodynamics of the TALE AvrBS3 binding to wild-type Bs3 and Bs3 A_0_, C_0_ and G_0_ target DNAs. (*a*) DNA sequence of the Bs3 DNA used in the fluorescence anisotropy and ITC binding assays (see also Supplementary Figs. S6 and S7). (*b*–*e*) DNA-binding profiles in the absence (solid circles) and the presence (open circles) of competitor DNA are depicted. The protein concentration [protein] has units of n*M*. The ITC assays show the nonlinear regression curve fitting of the data using a single-site binding model (solid circle) and a competitive binding model when the competitor DNA was present in the reaction (open circles). (*f*) The thermodynamic parameters of binding are reported. The obtained values are the average of four independent experiments. The *K*_A_ of AvrBs3 association is shown for comparison purposes. Asterisks indicate the values of *n*, *K*_A_ and Δ*H* obtained using a competitive-binding fitting model (see Supplementary Material). 1 cal = 4.186 J.

To characterize the thermodynamic parameters of the interactions between AvrBs3 and its target DNA, we quantitatively analyzed their association by fluorescence anisotropy (FA) and isothermal titration calorimetry (ITC) (Fig. 3 ▶). Oligonucleotides with different lengths containing the target sequences were initially tested (Supplementary Figs. S6 and S7), and the 21 bp probe was selected as the minimum binding length for specific recognition of the TALE AvrBs3 (Fig. 3 ▶ *a*). The *K*_d_ values measured by ITC display higher values by a factor of around 2–4 compared with the FA experiments. This difference is consistent with experimental variations and could arise from the physical properties measured in each approach, which require a different range of concentrations. However, despite these differences both techniques show the same tendencies for the same set of experiments. To examine the ability of the TALE AvrBs3 to discriminate between target DNA and other DNA sequences, we performed both FA and ITC experiments in the presence of a 24 bp non­specific DNA (see Supplementary Material).

The TALE AvrBs3 shows binding to the Bs3 duplex oligonucleotide with a dissociation constant of 33 n*M* by FA (Fig. 3 ▶ *b*). The TALE–DNA association is not affected when the affinity is measured in the presence of competitor DNA (*K*_d,FA_ = 36.5 n*M*; Fig. 3 ▶ *d*). The ITC binding measurements show the same behaviour (Figs. 3 ▶ *c* and 3 ▶ *e*). In addition, the ITC revealed that the protein–DNA association is exothermic (Δ*H* = −31.6 kcal mol^−1^) and the stoichiometry is close to one. The measurement of the reaction in the presence of competitor DNA (see *Materials and methods* [Sec sec2] and Figs. 3 ▶ *d* and 3 ▶ *e*) showed only minor variations in the thermodynamic parameters, indicating that the TALE AvrBs3 is able to bind its DNA target with high specificity in a spontaneous reaction (Fig. 3 ▶ *f*).

### 3.6. Functional relevance of the nucleotide in position 0

TALE proteins bind to the promoter regions enhancing and modulating the transcription of plant genes (Boch & Bonas, 2010 ▶). For example, the PthXo1 binding site is downstream of the TATA box, while the T at position 0 for the AvrBs3 target appears to be part of the TATA box. The initial T_0_ position in the target sequence has been reported to be an important nucleotide for TALE function (Boch *et al.*, 2009 ▶; Römer *et al.*, 2010 ▶) and for the binding of the protein to its target (Mahfouz *et al.*, 2011 ▶). The recognition of this nucleotide involves the less well conserved repeats −1 and 0. However, we did not observe direct interactions of these repeats with the base of T_0_ in the AvrBs3–DNA structure. A similar situation was detected in the PthXo1–DNA structure, in which T_0_ does not show direct interactions with the protein (Mak *et al.*, 2012 ▶). Instead, in AvrBs3 we observed a new conformation that allows the interaction of the N-terminal domain with T_0_ (Figs. 2 ▶ *b* and 2 ▶ *c*).

To address the preferences of AvrBs3 for the nucleotide at position 0 of its target DNA, we assessed its binding and thermodynamic parameters using Bs3 A_0_, G_0_ and C_0_ oligonucleotides in which T_0_ was substituted by the corresponding base (Figs. 3 ▶ *b*, 3 ▶ *c* and 3 ▶ *f*). TALE AvrBs3 binds Bs3 A_0_ and C_0_ with a similar *K*_d_ to the original T_0_. The presence of competitor DNA barely altered the affinity (Figs. 3 ▶ *d*, 3 ▶ *e* and 3*f* ▶). Only G_0_ displayed an increase of fourfold; however, the binding still showed a reasonable affinity that was hardly disturbed by the presence of competitor DNA. The ITC data support the FA binding measurements, although with small variations in the Δ*H* of the reaction. Thus, AvrBs3 is able to recognize and bind its DNA target with similar thermodynamic parameters when T_0_ is substituted by C_0_. When a bulkier base such as A is found at position 0, the *K*_d_ of the reaction is slightly higher using both methods (Figs. 3 ▶ *b*, 3 ▶ *c* and 3 ▶ *f*) and the Δ*H* of the reaction is less negative, indicating that even though the binding reaction is less efficient the presence of the larger base does not hamper binding. Only the presence of G_0_ showed an affinity decrease that did not impede target recognition even in the presence of competitor DNA (Figs. 3 ▶ *b*–3 ▶ *f*). Hence, *in vitro* AvrBs3 can bind its target sequence including the C_0_, G_0_ and A_0_ mutations with an affinity similar to the wild-type DNA target. The presence of a bulkier base at the 0 position does not seem to hamper binding to the AvrBs3 target *in vitro*, which is in agreement with the protein–DNA interactions in this region, which involve only the DNA backbone.

**Figure 4:**
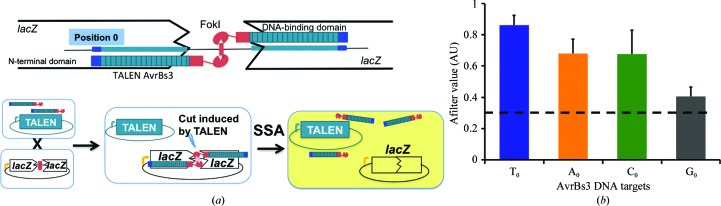
(*a*) Principle of the yeast screening assay. A strain harbouring an expression vector encoding a TALEN is mated with a strain harbouring a reporter plasmid. The reporter plasmid bears a *lacZ* reporter gene interrupted by an insert containing a TALEN target site flanked by two direct repeats. Upon mating, the TALEN generates a double-strand break at the site of interest, allowing the restoration of a functional *lacZ* gene by SSA and enabling the generation of a blue colour in the presence of X-Gal. The colour was quantified and scored as the Afilter value, a parameter correlated to TALEN nuclease activity. (*b*) Nuclease activity of the AvrBs3 TALEN towards Bs3 homodimeric targets harbouring either T, A, C or G at position 0. Afilter values obtained with the four different Bs3 homodimeric targets are displayed. The dashed line indicates the experimental background level. The obtained values are the average of three independent experiments.

To analyze this effect *in vivo*, we used a single-strand annealing assay (SSA) to assess whether changes at the zero position of the target sequence could affect the binding of an AvrBs3 fused to a FokI nuclease domain, disturbing its activity (see *Materials and methods* [Sec sec2]; Arnould *et al.*, 2007 ▶; Cermak *et al.*, 2011 ▶; Grizot *et al.*, 2009 ▶; Fig. 4 ▶ *a*). The homodimeric Bs3 target sequence containing either T_0_, A_0_, C_0_ or G_0_ was inserted into an episomal plasmid to assess the preference of AvrBs3 for the nucleotide at position 0. The assay showed that AvrBs3–FokI (TALEN) was able to target its site independently of the nucleotide at position 0. Although the T_0_-containing target displays the higher activity (Fig. 4 ▶ *b*), our results suggest that T_0_ substitution does not seem to exhibit a large effect in TALE binding and only the G_0_ target, with the bulkiest base, displayed a marked decrease in activity, in agreement with the *in vitro* binding measurements, suggesting that this approach could be employed to estimate the efficiency of *in vivo* applications. Functional analysis in engineered TALE activating the endogenous human NTF3 gene has also shown that constructs containing repeats −1 and 0 can target DNA sequences lacking T_0_ *in vivo* (Miller *et al.*, 2011 ▶). Hence, our *in vitro* data suggest that T_0_ may not play an essential role in DNA binding of the TALE AvrBs3, increasing the number of DNA sequences that could be targeted by this scaffold. A possible explanation for the prevalence of T at position zero in the natural TALE targets could be attributed to the AT-rich sequence within the promoter region rather than to selective recognition of the nucleotide at this position.

## 4. Concluding remarks

The assembly of a redesigned TALE recognizing new DNA targets has confirmed the modularity of these DNA-binding domains to engineer new specificities. This property makes this scaffold a good candidate to tailor devices that when fused with other catalytic domains, such as nucleases, methylases acetylases *etc.*, could shuttle to specific genome loci a determined activity for controlling gene expression, genome modification or gene repair (Prieto *et al.*, 2012 ▶). However, it is not clear whether the binding mechanism depends on a minimum number of perfect matches within a given DNA target length or perhaps involves differential contributions of different associations between the RVDs and the nucleotides (Boch *et al.*, 2009 ▶; Streubel *et al.*, 2012 ▶). In this context, activity assays have supported the important influence of T_0_ in target recognition (Boch *et al.*, 2009 ▶). Only the presence of a purine influences target binding. This effect is emphasized in the case of guanine, the bulkiest base. Therefore, other nucleotides could be accommodated in this position, thus increasing the number of target sequences that may be engineered in this scaffold.

## Supplementary Material

PDB reference: AvrBs3–DNA complex, 2ypf

Supplementary material file. DOI: 10.1107/S0907444913016429/yt5053sup1.pdf

## Figures and Tables

**Table 1:** Data-collection and refinement statistics Values in parentheses are for the highest resolution shell.

	Native	Ta_6_Br_12_ peak SAD
Data collection		
Space group	*C*2	*C*2
Unit-cell parameters (Å, °)	*a* = 151.11, *b* = 100.25, *c* = 61.37, β = 102.55	*a* = 152.27, *b* = 100.53, *c* = 61.40, β = 102.84
Resolution (Å)	32.31–2.55 (2.61–2.55)	50–3.70 (3.90–3.70)
*R*_merge_ †	0.044 (0.62)	0.041 (0.23)
No. of reflections	29077	19214
〈*I*/σ(*I*)〉	11.79 (1.31)	8.27 (2.2)
Completeness (%)	93.26 (95.1)	97.6 (94.7)
Multiplicity	2.22 (2.07)	2.9 (2.8)
Wavelength (Å)	1.00	1.254
Beamline	SLS-XS06A	SLS-XS06A
Refinement		
Resolution (Å)	32.29–2.55	
No. of reflections	25764	
*R*_work_/*R*_free_	0.24/0.29	
No. of atoms		
Protein	4777	
Nucleic acid	874	
Water	180	
R.m.s. deviations		
Bond lengths (Å)	0.012	
Bond angles (°)	1.897	
Average *B* factor (Å^2^)	72.2	
Ramachandran plot, residues in (%)		
Allowed regions	92.85	
Generously allowed regions	6.27	
Disallowed regions	0.98	

† *R*_merge_ is defined according to *XDS* (Kabsch, 2010 ▶).

## Footnotes

1 Supplementary material has been deposited in the IUCr electronic archive (Reference: YT5053). Services for accessing this material are described at the back of the journal.
