# Supplementary material for: Structure of the AvrBs3–DNA complex provides new insights into the initial thymine-recognition mechanism
Source: Acta Crystallogr D Biol Crystallogr. 2013 Aug 15;69(Pt 9):1707–16. doi: 10.1107/S0907444913016429 (PMC3760130; doi:10.1107/S0907444913016429)
Supplement: Supplementary file 1 [file d-69-01707-sup1.pdf]

**N-t**		**152**	VDLRTLGYSQQQQEKIKPKVRSTVAQHHEALVGHGFTHAHIVALSQHPAALGTVAVKYQDMIAALPEATHEA
**T**	-1	**225**	I V G V G K Q W S G **A R** A L E A L L T V A G E L R G P P L Q
**T**	0	**255**	L D T G Q L L K I A **K R** G G V T A V E A V H A W R N A L T G A P L N
**A**	1	**289**	L T P E Q V V A I A S **H D** G G K Q A L E T V Q R L L P V L C Q A H G
**T**	2	**323**	L T P Q Q V V A I A S **N G** G G K Q A L E T V Q R L L P V L C Q A H G
**A**	3	**357**	L T P Q Q V V A I A S **N I** G G K Q A L E T V Q R L L P V L C Q A H G
**T**	4	**391**	L T P E Q V V A I A S **N G** G G K Q A L E T V Q R L L P V L C Q A H G
**A**	5	**425**	L T P E Q V V A I A S **N I** G G K Q A L E T V Q A L L P V L C Q A H G
**A**	6	**459**	L T P E Q V V A I A S **N I** G G K Q A L E T V Q A L L P V L C Q A H G
**A**	7	**493**	L T P E Q V V A I A S **N I** G G K Q A L E T V Q A L L P V L C Q A H G
**C**	8	**527**	L T P E Q V V A I A S **H D** G G K Q A L E T V Q R L L P V L C Q A H G
**C**	9	**561**	L T P E Q V V A I A S **H D** G G K Q A L E T V Q R L L P V L C Q A H G
**T**	10	**595**	L T P Q Q V V A I A S **N G** G G K Q A L E T V Q R L L P V L C Q A H G
**A**	11	**629**	L T P E Q V V A I A S **N I** G G K Q A L E T V Q A L L P V L C Q A H G
**A**	12	**663**	L T P E Q V V A I A S **N I** G G K Q A L E T V Q R L L P V L C Q A H G
**C**	13	**697**	L T P E Q V V A I A S **H D** G G K Q A L E T V Q R L L P V L C Q A H G
**C**	14	**731**	L T P E Q V V A I A S **H D** G G K Q A L E T V Q R L L P V L C Q A H G
**C**	15	**765**	L T P E Q V V A I A S **H D** G G K Q A L E T V Q R L L P V L C Q A H G
**T**	16	**799**	L T P Q Q V V A I A S **N G** G G R P A L E T V Q R L L P V L C Q A H G
**C**	17	**833**	L T P E Q V V A I A S **H D** G G K Q A L E T V Q R L L P V L C Q A H G
**T**	$17_{1/2}$	**867**	L T P Q Q V V A I A S **N G** G G R P A E S I V A Q L S R P D P **895**

Supp. Figure 1.- TALE AvrBs3 protein sequence. The numbering follows the order in the wild type AvrBs3 protein. The RVDs are depicted in bold.

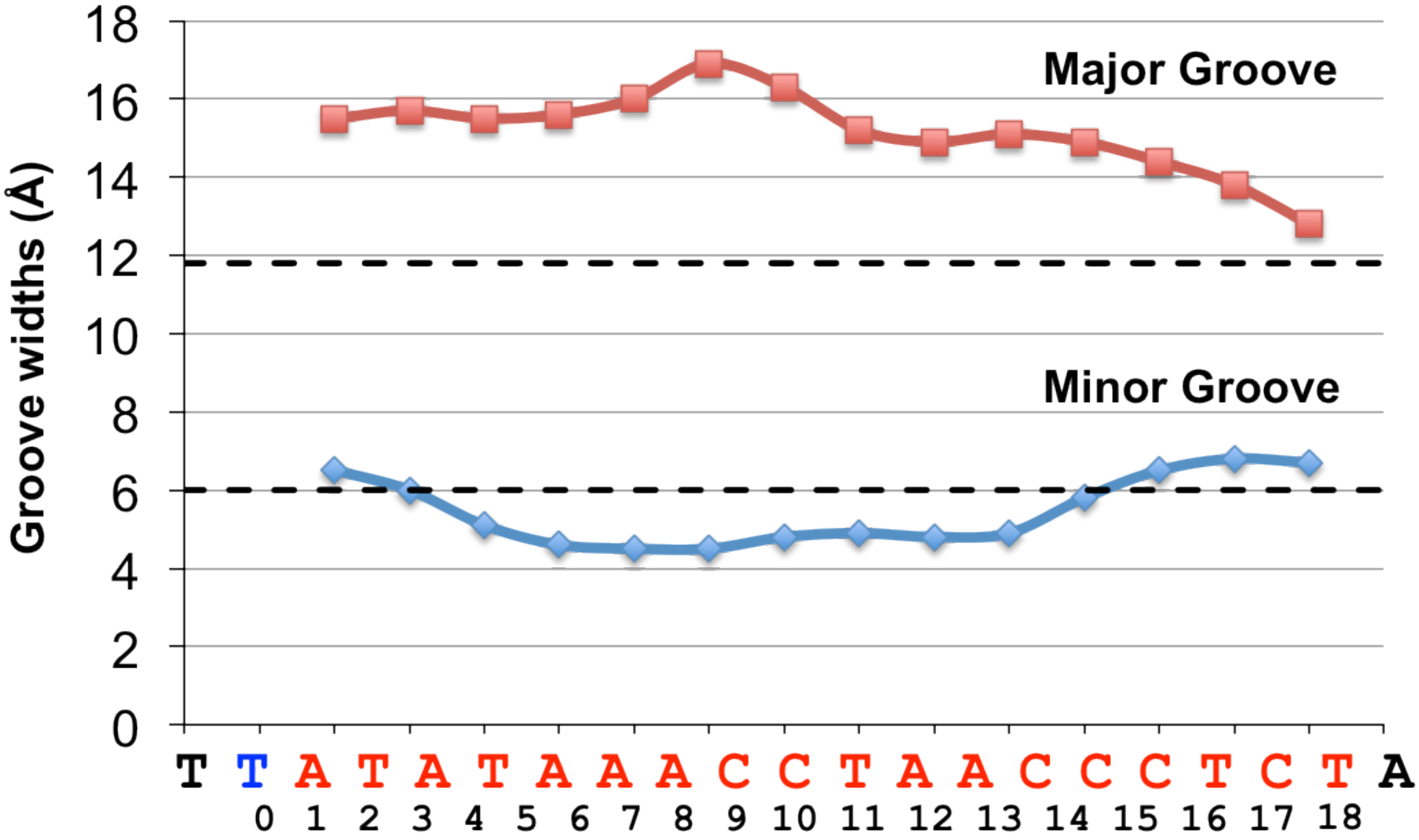

Supp. Figure 2.- DNA width. The minor and major groove DNA width have been determined by 3DNA analysis Lu & Olson (2003) *Nucleic Acids Res.* **31(17)**, 5108-21. Dashed lines indicate the canonical groove width for B-DNA.

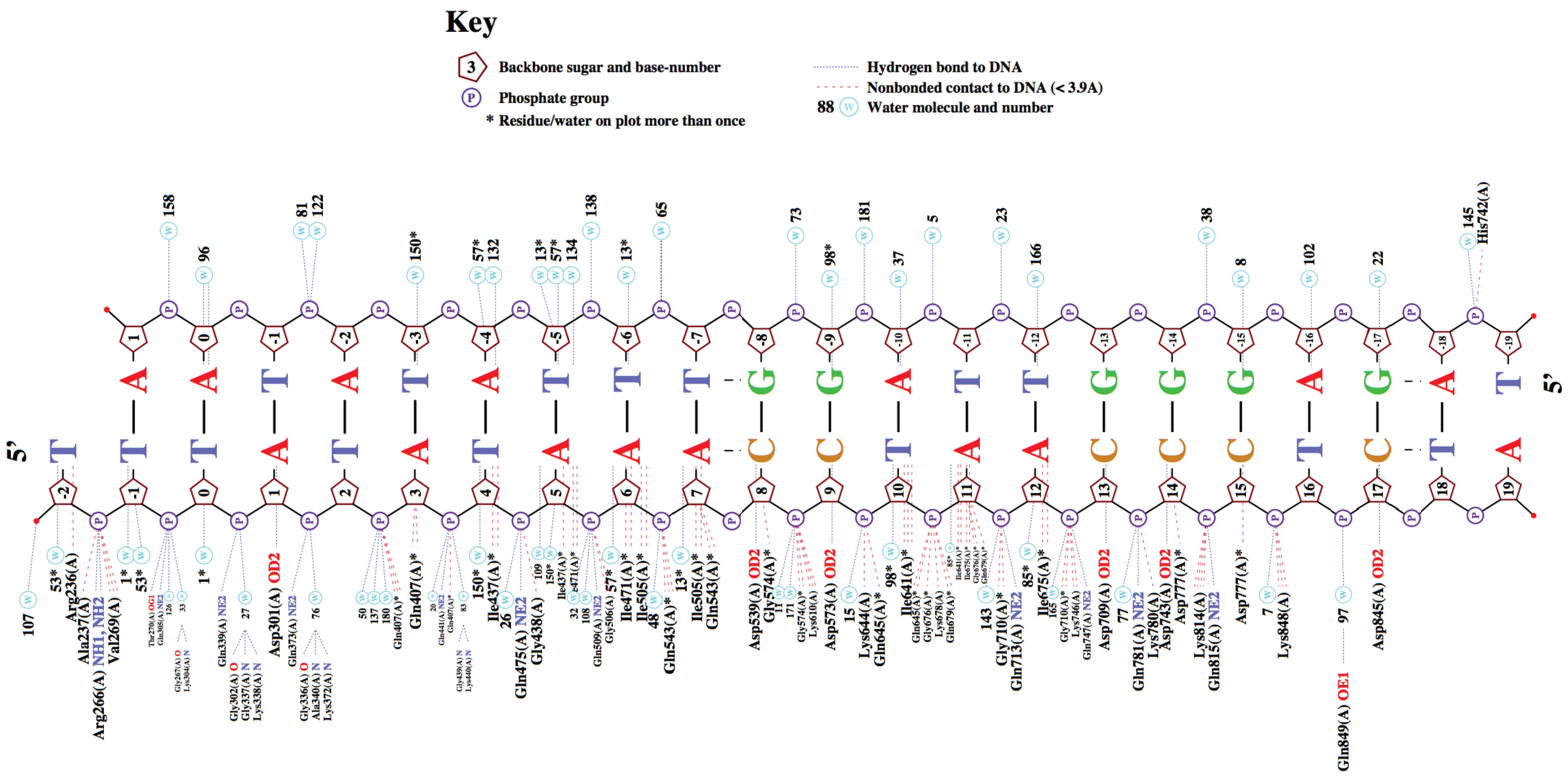

Supp. Figure 3.- Scheme of the Protein-DNA contacts in the AvrBs3 crystal structure. Lines in blue and red indicate hydrogen bonds and van der Waals interactions, respectively. Blue dots represent water molecules involved in the interaction. The scheme has been generated with NUCPLOT: a program to generate schematic diagrams of protein-DNA interactions. Luscombe N M, Laskowski R A, Thornton J M (1997). *Nucleic Acids Res., 25, 4940-4945.*

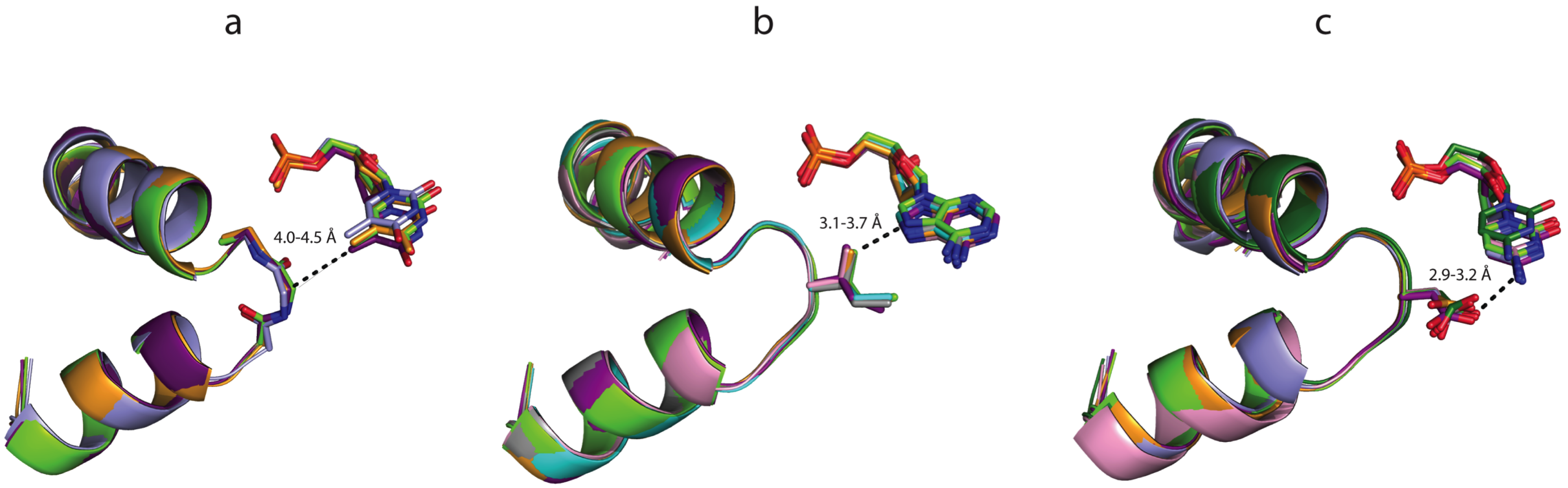

Supp. Figure 4.- Contacts between the TALE AvrBs3 and the DNA bases. Four combinations of RDVs and nucleotide are present in the TALE AvrBs3 and Bs3 target DNA complex. HD to adenine repeat (see Figure2a and main text). a) NG to T in the 2nd, 4th, 10th and 16th repeats. The glycine allows enough space for the placement of the methyl group of the thymine. b) NI to adenine in the 3rd, 5th, 6th, 7th, 11th and 12th repeats. The isoleucine contacts through van der Waals interactions with the base. c) HD to cytosine in the 8th, 9th, 13th, 14th, 15th and 17th repeats. The aspartic acid contacts by hydrogen bond the cytosine.

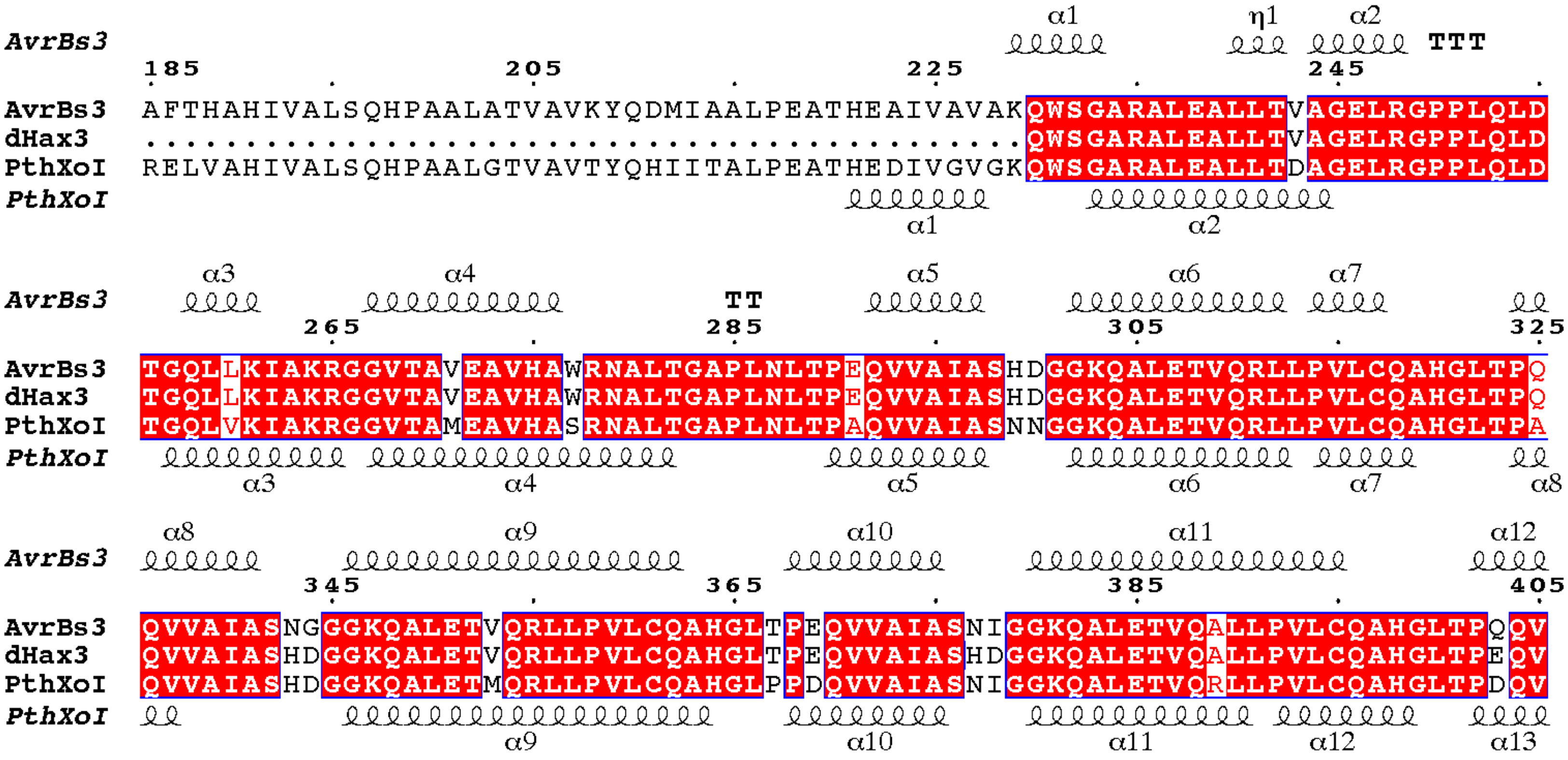

Supp. Figure 5.- Sequence alignment of the N-terminal region from the available TALE structures. The figure was obtained using (http://espript.ibcp.fr/ESPript/ESPript/). The alignment was performed with ClustalW (http://www.ebi.ac.uk/Tools/msa/clustalw2/) and the secondary structures of AvrBs3 (top sketch) and PthXoI (bottom sketch) were calculated using DSSP (Kabsch,W. and Sander,C. 1983 Biopolymers 22, p2577-2637) and superimposed on the aligned sequences for comparison.

Supp. Figure 6.- Fluorescence Anisotropy assays using a 19 and 23 bp DNA including the Bs3-DNA target sequence. The Kds of the interactions in the presence and in the absence of competitor are reported in the tables. The lower panels show the DNA binding profiles.

19bp Bs3

TATATAAACCTAACCCTCT

	AvrBs3	AVrBs3+ comp DNA
Kd (nM)	389.5±20.8	540.5±56

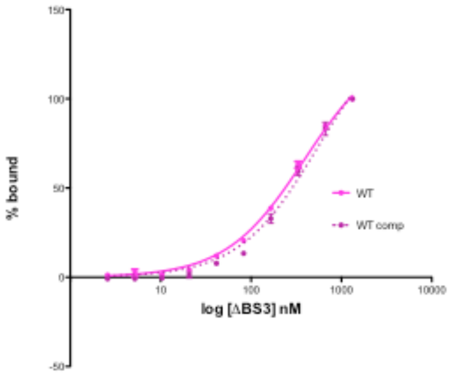

23bp Bs3

ATTATATAAACCTAACCCTCTAT

	AvrBs3	AVrBs3+ comp DNA
Kd (nM)	61.6±6	67.9±6

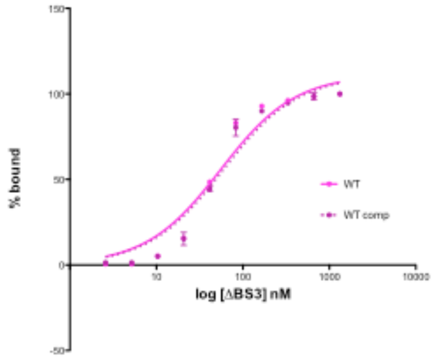

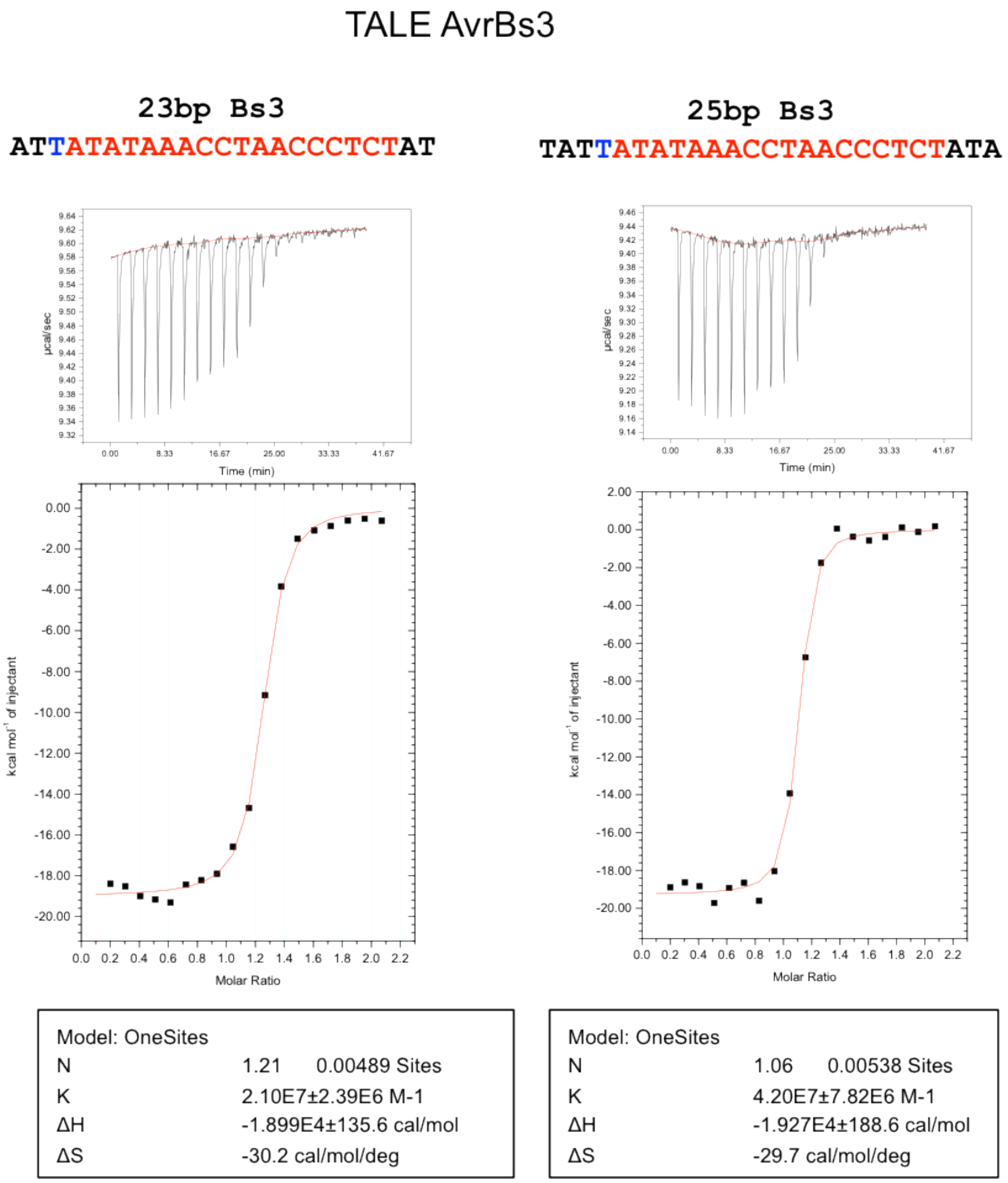

Supp. Figure 7.- ITC measurements using the 23 and 25bp DNA duplexes containing the Bs3 sequence. Top panels show the raw data of the binding isotherm of TALE AvrBs3. The middle panel shows the non-linear regression curve fitting of the data by using one-site binding model. The thermodynamic parameters are reported in the bottom panels.
